# Hormesis: A New Religion?

**DOI:** 10.1289/ehp.114-1665404

**Published:** 2006-11

**Authors:** Kristina A. Thayer, Ronald Melnick, James Huff, Kathy Burns, Devra Davis

**Affiliations:** National Institute of Environmental Health Sciences, National Institutes of Health, Department of Health and Human Services, Research Triangle Park, North Carolina, E-mail: thayer@niehs.nih.gov; Sciencecorps, Lexington, Massachusetts; University of Pittsburgh, Graduate School of Public Health, Department of Epidemiology, Center for Environmental Oncology, University of Pittsburgh Cancer Institute, Pittsburgh, Pennsylvania

Cook and Calabrese (2006) make inaccurate claims about our perspective on hormesis (Thayer et al. 2005). They define hormesis as “low-dose stimulation and high-dose inhibition,” declaring “beneficial/ harmful effects should not be part of the definition, but reserved to subsequent evaluation. . . .” Yet, they advocate higher permissible environmental levels of hazardous agents based on purported health benefits. Cook and Calabrese promote changing the way carcinogens are regulated to accommodate hormesis, recognizing that this “would result in cancer risk assessment values about 100- to 200-fold higher than currently employed” (Calabrese and Cook 2005). Previously, Calabrese and Baldwin (2003a) stated, “agencies will need to accept the possibility (actually, the likelihood) that toxic substances, even the most highly toxic (e.g., cadmium, lead, mercury, dioxin, PCBs, etc.) can cause beneficial effects at low doses.”

We are concerned that changing health policies to permit higher exposures based on alleged benefits would be harmful, particularly to susceptible subgroups and individuals exposed to mixtures (Thayer et al. 2005). Instead Cook and Calabrese (2006) suggest that policy decision making “may tend to bring various subgroups in the population together to debate one group’s health benefit against another group’s health risk.” To pit one group against another is absurd. Health-protective default assumptions that are used to compensate for uncertainties should not be dismissed based on untested propositions that likely incur greater risks.

Contrary to statements made by Cook and Calabrese (2006), in our article (Thayer et al. 2005) we never claimed that hormetic responses are rare. Rather, we argued that hormesis should not be assumed as universal. In fact, we have published on nonmonotonic dose responses in biological systems (Kohn and Melnick 2002; Welshons et al. 2003). We argue against the assumption that “an exposure limit in the range of the maximum stimulation could promote appreciable benefits in public health” for the general population (Cook and Calabrese 2006). Yet, we fully support addressing non-monotonic dose–response relationships in risk assessments.

Further, we never claimed that “comprehensive mechanistic knowledge is necessary” before making a public health decision. In fact, we have a history of arguing the contrary. Indeed, if this standard were operating today, we might still be debating the dangers of tobacco smoke and benzene, among many others. Calabrese appears to overstate the frequency of hormetic dose–response curves. Some responses considered “stimulatory” are not, such as decreased interleukin-2 release, blood pressure, memory, and prolactin level (Calabrese and Baldwin 2003b). His hormesis database contains U- or J-shaped curves where the low dose “stimulation” is actually decreased compared to control values (Calabrese and Baldwin 2001). There should be some mechanistic indication of what specifically is being stimulated (and inhibited at higher doses) before considering a curve hormetic. Otherwise, the empirical observations of Calabrese and colleagues simply reflect nonmonotonic dose responses.

The quote we used from the BEIR VII (National Research Council 2005) that draws attention to the lack of evidence of a health benefit from low doses of ionizing radiation was not misleading; Kaiser (2005) also reported that the National Research Council dismissed “the hypothesis that tiny amounts of [ionizing] radiation are harmless or even beneficial,” noting that cancer risk increases proportionally with exposure. In contrast, Calabrese and Cook (2005) claimed that all or most carcinogens have a hormetic dose(s) at which tumors will be decreased. This is contrary to what we know about the carcinogenicity of chemicals and radiation.

Labeling a dose response as hormetic to justify higher exposures and claimed benefits for the general population without providing scientific evidence is counter to public-health protective assumptions. For example, cadmium has been touted as a hormetic agent with benefits (Calabrese and Baldwin 2003) because low doses are associated with decreases in testicular tumors in rats. However, Waalkes et al. (1997, 1988) reported increases in prostate tumors within the hormetic dose range for testicular tumors. In our article (Thayer et al. 2005), we emphasized the latter, whereas it was seemingly ignored by Calabrese and Baldwin (2003), because cadmium is a human carcinogen and includes associations with cancer of the prostate and other organs [National Toxicology Program (NTP) 2004]. In addition, differential susceptibility must be addressed because it is well established that cancer and other health risks from ionizing radiation, some chemotherapeutics, and passive tobacco smoke are much greater for those exposed *in utero* or as children. We should not allow another tragedy such as the one caused by diethylstilbestrol.

Disease prevention strategies should not rely on higher environmental exposures to known toxicants (e.g., cadmium, lead, mercury, dioxin, polychlorinated biphenyls). Setting environmental exposure limits based on ranges of maximum stimulation (i.e., equated with postulated hormetic benefits) is a totally unjustified public health policy that would impose greater involuntary risks on sizable segments of the population.

## References

[b1-ehp0114-a00632] Calabrese EJ, Baldwin LA (2001). The frequency of U-shaped dose responses in the toxicological literature. Toxicol Sci.

[b2-ehp0114-a00632] Calabrese EJ, Baldwin LA (2003a). Hormesis: the dose-response revolution. Annu Rev Pharmacol Toxicol.

[b3-ehp0114-a00632] Calabrese EJ, Baldwin LA (2003b). Peptides and hormesis. Crit Rev Toxicol.

[b4-ehp0114-a00632] Calabrese EJ, Cook RR (2005). Hormesis: how it could affect the risk assessment process. Hum Exp Toxicol.

[b5-ehp0114-a00632] Cook R Calabrese EJ. 2006. The importance of hormesis to public health. Environ Health Perspect 1141631–1635; 10.1289/ehp.8606 [Online 10 July 2006] 10.1289/ehp.8606 PMC1665397 17107845

[b6-ehp0114-a00632] Kaiser J (2005). Epidemiology. Radiation dangerous even at lowest doses. Science.

[b7-ehp0114-a00632] Kohn MC, Melnick RL (2002). Biochemical origins of the non-monotonic receptor-mediated dose-response. J Mol Endocrinol.

[b8-ehp0114-a00632] National Research Council 2005. Health Risks from Exposure to Low Levels of Ionizing Radiation: BEIR VII Phase 2. Washington, DC:National Academies Press. Available: http://www.nap.edu/books/030909156X/html [accessed 18 August 2005].

[b9-ehp0114-a00632] NTP 2004. Report on Carcinogens. Eleventh Edition. Research Triangle Park, NC:National Toxicology Program. Available: http://ntp.niehs.nih.gov/go/19914 [accessed 25 August 2006].

[b10-ehp0114-a00632] Thayer KA, Melnick R, Burns K, Davis D, Huff J (2005). Fundamental flaws of hormesis for public health decisions. Environ Health Perspect.

[b11-ehp0114-a00632] Waalkes MP, Rehm S, Devor DE (1997). The effects of continuous testosterone exposure on spontaneous and cadmium-induced tumors in the male Fischer (F344/NCr) rat: loss of testicular response. Toxicol Appl Pharmacol.

[b12-ehp0114-a00632] Waalkes MP, Rehm S, Riggs CW, Bare RM, Devor DE, Poirier LA (1988). Cadmium carcinogenesis in male Wistar [Crl:(WI)BR] rats: dose-response analysis of tumor induction in the prostate and testes and at the injection site. Cancer Res.

[b13-ehp0114-a00632] Welshons WV, Thayer KA, Judy BM, Taylor JA, Curran EM, vom Saal FS (2003). Large effects from small exposures. I. Mechanisms for endocrine-disrupting chemicals with estrogenic activity. Environ Health Perspect.

